# Transcriptome Analysis of an *Aedes albopictus* Cell Line Single- and Dual-Infected with Lammi Virus and WNV

**DOI:** 10.3390/ijms23020875

**Published:** 2022-01-14

**Authors:** Pontus Öhlund, Nicolas Delhomme, Juliette Hayer, Jenny C. Hesson, Anne-Lie Blomström

**Affiliations:** 1 Department of Biomedical Sciences and Veterinary Public Health, Swedish University of Agricultural Sciences, Box 7028, 750 07 Uppsala, Sweden; anne-lie.blomstrom@slu.se; 2 Umeå Plant Science Centre (UPSC), Department of Forest Genetics and Plant Physiology, Swedish University of Agricultural Sciences, 901 83 Umea, Sweden; nicolas.delhomme@slu.se; 3 MIVEGEC, IRD, CNRS, University of Montpellier, 34394 Montpellier, France; juliette.hayer@ird.fr; 4 SLU-Global Bioinformatics Centre, Department of Animal Breeding and Genetics, Swedish University of Agricultural Sciences, Box 7023, 750 07 Uppsala, Sweden; 5 Department of Medical Biochemistry and Microbiology/Zoonosis Science Center, Uppsala University, Box 582, 751 23 Uppsala, Sweden; jenny.hesson@imbim.uu.se

**Keywords:** insect-specific flaviviruses, West Nile virus, *Aedes albopictus*, transcriptome, viral interference

## Abstract

Understanding the flavivirus infection process in mosquito hosts is important and fundamental in the search for novel control strategies that target the mosquitoes’ ability to carry and transmit pathogenic arboviruses. A group of viruses known as insect-specific viruses (ISVs) has been shown to interfere with the infection and replication of a secondary arbovirus infection in mosquitoes and mosquito-derived cell lines. However, the molecular mechanisms behind this interference are unknown. Therefore, in the present study, we infected the *Aedes albopictus* cell line U4.4 with either the West Nile virus (WNV), the insect-specific Lammi virus (LamV) or an infection scheme whereby cells were pre-infected with LamV 24 h prior to WNV challenge. The qPCR analysis showed that the dual-infected U4.4 cells had a reduced number of WNV RNA copies compared to WNV-only infected cells. The transcriptome profiles of the different infection groups showed a variety of genes with altered expression. WNV-infected cells had an up-regulation of a broad range of immune-related genes, while in LamV-infected cells, many genes related to stress, such as different heat-shock proteins, were up-regulated. The transcriptome profile of the dual-infected cells was a mix of up- and down-regulated genes triggered by both viruses. Furthermore, we observed an up-regulation of signal peptidase complex (SPC) proteins in all infection groups. These SPC proteins have shown importance for flavivirus assembly and secretion and could be potential targets for gene modification in strategies for the interruption of flavivirus transmission by mosquitoes.

## 1. Introduction

Mosquitoes serve as vectors for many viral pathogens, and their associated diseases cause a major health burden across the globe. Flaviviruses, such as the West Nile virus (WNV), Dengue virus (DENV) and Zika virus (ZIKV), impose huge burdens on human and animal health [1,2,3]. For many of these arboviruses, there are no preventive vaccines or therapeutic drugs available and the reduction in transmission relies on traditional methods, e.g., suppressing mosquito populations with insecticides [4]. However, this is very cumbersome and costly, and, with the increase in insecticide resistance, these strategies are becoming less effective [5]. Novel control strategies are needed and the development of transgenic mosquitoes for either population suppression or population replacement with reduced vector competence (the ability of a mosquito to carry and transmit a virus) are among the proposed methods [6,7]. However, to find gene modification targets that interfere with the transmission of viral pathogens, we must first study the mechanisms that contribute to vector competence and viral dissemination in the mosquito vector.

Most mosquito-borne viruses persist in nature through transmission cycles between the mosquito and a vertebrate reservoir [8]. Once the mosquito has taken up the virus through a blood meal and has subsequently been infected, the innate antiviral immune response mounts an array of molecular signaling pathways and immune effector proteins to control the infection. Mosquitoes lack an adaptive immunity, and the main immune pathways are thought to be the RNA interference (RNAi) pathway, the toll pathway, the immune deficiency (Imd) pathway and the Janus kinase-signal transducer (JAK/STAT) pathway [9,10,11]. The RNAi pathway generates small RNAs from viral double-stranded RNA which are used as template to target and degenerate complementary viral RNA, therefore inhibiting viral replication [12]. The other three are signaling pathways activated upon recognition of pathogen-associated molecular patterns (PAMPs) by pattern-recognition receptors (PRRs), which trigger a signaling cascade leading to the production of effector proteins such as antimicrobial peptides (AMPs) [13]. There are several PRRs in mosquitoes, including peptidoglycan-recognition protein, fibrinogen-related proteins, scavenger receptors and C-type lectins. PRRs play important roles by binding invading pathogens and mediating immune responses, such as melanization and phagocytosis [14,15]. AMPs are potent immune-effectors with antimicrobial activities and consist of several classes, such as defensins, cecropins, gambicin, diptericin and attacins, categorized based on their structure, function and specificity [16,17,18,19,20]. Other important immune proteins in insects are the serine proteases, such as the CLIP proteases; these are present in the hemolymph and act in cascade pathways initiated by different stimuli, such as PAMPs. Activated CLIP-C cleaves and activates CLIP-B, which, in turn, cleaves and activates the toll-ligand Spätzle or prophenoloxidase, which is required for the melanization response [21].

Gene expression profiling in response to arbovirus infection in mosquitoes and mosquito-derived cell lines has revealed not only the alteration of immune-related genes, but also biological processes, such as metabolic pathways, stress, translation and more. Transcriptomics studies have been conducted to investigate the response for major arboviruses such as WNV, ZIKV, DENV and Chikungunya virus (CHIKV) [22,23,24,25,26,27,28]. However, mosquitoes are often naturally and persistently infected with a group of viruses that are known as insect-specific viruses (ISVs) [29]. This group of viruses is unable to infect vertebrates and are maintained in the mosquito population through vertical transmission, from mother to offspring [30,31,32,33]. ISVs are interesting for several reasons; one is that many of these viruses are thought to be ancestors to the pathogenic arboviruses [34]. Another reason is that a primary infection of certain ISVs can block or interfere with a secondary infection of a pathogenic arbovirus. The insect-specific Palm Creek virus has been shown to reduce infection and replication of WNV [35,36], while similar results were observed with the insect-specific Nhumirim virus [37,38]. The Lammi virus (LamV) is an insect-specific flavivirus (ISFV) that was first isolated from *Aedes cinereus* mosquitoes in Finland [39]. LamV is phylogenetically affiliated with the medically important dual-host flaviviruses, but presents the insect-restriction phenotype and is referred to as dual-host affiliated ISFV [40]. Recently, we have shown that LamV induces a strong small RNA response in the *Aedes albopictus* cell line U4.4 [41].

In the present study, we analyzed the transcriptomic data of *Ae. albopictus* cells infected with either WNV, insect-specific LamV or an infection scheme whereby cells were pre-infected with LamV prior to challenge with WNV. Our aim is to improve the understanding of the ISFV interaction and its effects on the host. Furthermore, we investigated the possible interfering effects of a prior LamV infection on WNV replication.

## 2. Results

RNA-sequencing was conducted on poly(A)-enriched RNA extracted from the *Ae. albopictus* cell line U4.4 infected with either WNV, insect-specific LamV or an infection scheme whereby cells were pre-infected with LamV 24 h before being challenged with WNV. Cells from the different infection groups, including mock-infected cells, were collected between 24 and 72 hpi as biological triplicates. A total of 30 RNA-seq libraries were created, generating between 31.1 and 50.9 million paired-end reads per sample. The data were of good quality and the samples clustered as expected when analyzed with a principal component analysis (PCA) (Figure S1).

### 2.1. qPCR Results of Supernatant

**Figure 1:**
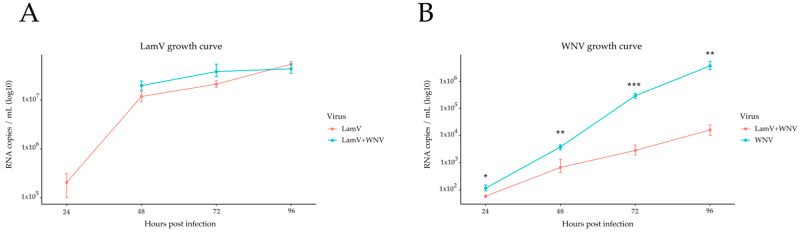
Growth curves of LamV and WNV in U4.4 cells, shown as RNA copies/mL over time. (**A**) LamV growth curve. (**B**) WNV growth curve. Dots show the mean RNA copy number per mL with a standard deviation among the biological replicates. * *p* < 0.05, ** *p* < 0.01, *** *p* < 0.001.

To investigate the possible interfering effect of LamV on WNV replication, RNA from the supernatant was used to follow the infection over time (Figure 1). The results show that the level of LamV RNA copies were similar between cells infected only with LamV and cells challenged with WNV (Figure 1A). However, the level of WNV RNA copies were significantly lower in those cells pre-infected with LamV than in cells infected with WNV only (Figure 1B). The statistical significance differences were calculated using Student’s *t*-test.

### 2.2. Transcripts Differentially Expressed (DE) in LamV-Infected U4.4 Cells

**Figure 2:**
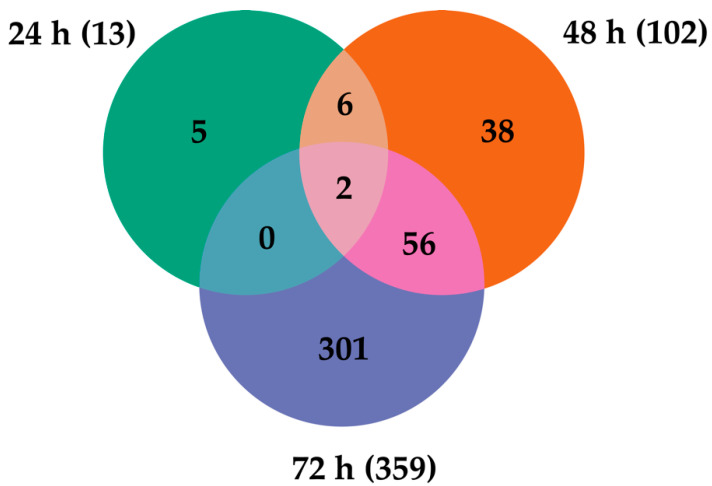
Venn diagram representing the number of differentially expressed transcripts at the different time points post-infection with LamV.

**Figure 3:**
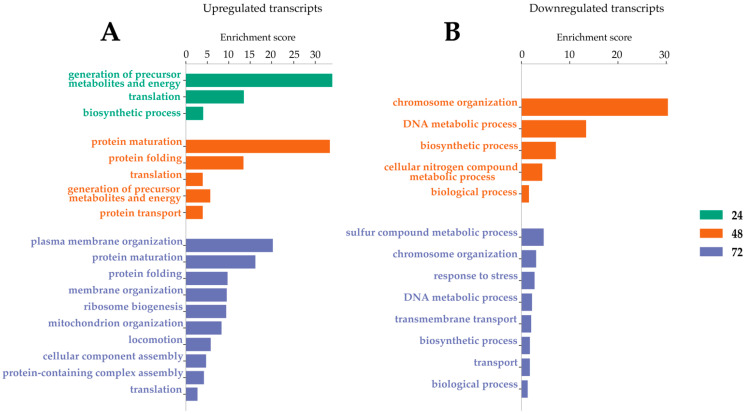
Gene ontology enrichment analysis for biological processes after LamV infection. (**A**) GO slim terms for up-regulated transcripts. (**B**) GO slim terms for down-regulated transcripts.

The analysis and comparison of mRNA expression profiles of *Ae. albopictus* U4.4 cells at different time points following LamV infection revealed that 13 (13 up-regulated), 102 (84 up- and 18 down-regulated) and 359 (180 up- and 179 down-regulated) transcripts were significantly differentially expressed (DE) at 24 h, 48 h and 72 hpi, respectively. The comparison of the transcriptome profiles showed two overlapping transcripts among all time points, both coding for the 40S ribosomal protein S21—AALF005471 (7.08–11.38-fold change) and AALF002626 (8–10.94-fold change) (Figure 2 and Table S1). Among the 56 overlapping transcripts between the two later time points (Figure 2 and Table S1), we observed an up-regulation of genes related to protein processes in the endoplasmic reticulum (ER), such as BiP/GRP78 (AALF021835), lethal(2) essential for life protein (AALF005663 and AALF015016), endoplasmin (AALF011939) and Signal peptidase complex subunit 3 (AALF003192). Among the down-regulated transcripts, we observed genes related to DNA replication, such as three DNA helicases (AALF015599, AALF022735 and AALF020651), and DNA replication licensing factor MCM7 (AALF006549). The GO enrichment analysis for biological processes resulted in three, five and ten GO slim terms for the up-regulated transcripts at 24 hpi, 48 hpi and 72 hpi, while the down-regulated transcripts at 48 hpi and 72 hpi resulted in five and eight GO slim terms (Figure 3).

### 2.3. Transcripts Differentially Expressed in WNV-Infected U4.4 Cells

**Figure 4:**
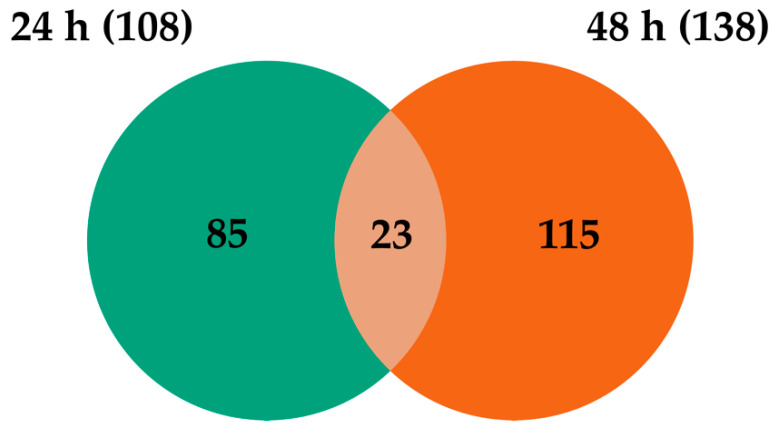
Venn diagram representing the number of differentially expressed transcripts at the different time points post-infection with WNV.

**Figure 5:**
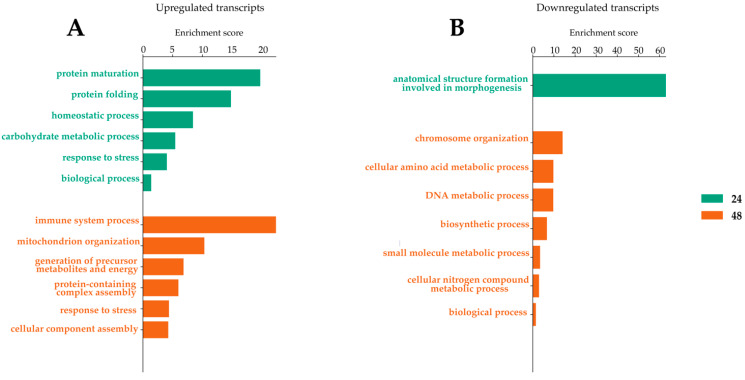
Gene ontology enrichment analysis for biological processes after WNV infection. (**A**) GO Slim terms for up-regulated transcripts. (**B**) GO slim terms for down-regulated transcripts.

When comparing WNV-infected U4.4 cells to mock controls, there were 108 (65 up- and 43 down-regulated) and 138 (85 up- and 53 down-regulated) transcripts that were significantly DE at 24 hpi and 48 hpi. The comparison of the transcriptome profiles showed 23 overlapping transcripts that were DE at both time points (Figure 4). Among these transcripts, many of the up-regulated genes were related to immune activity, such as C-type lectin (AALF016234), clip-domain serine protease family B (AALF019859), Defensin (AALF008821) and peptidoglycan-recognition protein (AALF020799) (Table 1 and Table S2). The GO enrichment analysis for biological processes of the up- and down-regulated genes at 24 hpi and 48 hpi reveled six different GO slim terms for the up-regulated genes and one and seven GO slim terms for the down-regulated transcripts (Figure 5).

### 2.4. Transcripts Differentially Expressed in Dual-Infected U4.4 Cells

**Figure 6:**
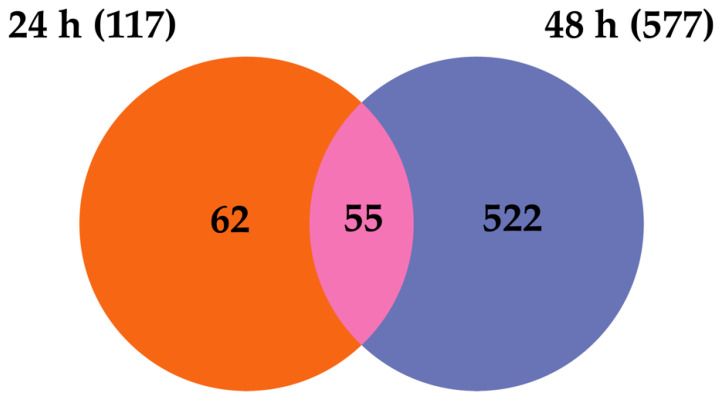
Venn diagram representing the number of differentially expressed transcripts at the different time points of dual-infected U4.4 cells. Timepoints 24 h and 48 h indicate time post-infection with WNV.

**Figure 7:**
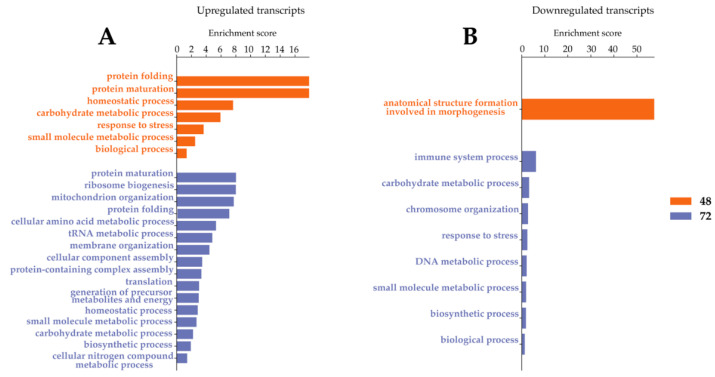
Gene ontology enrichment analysis for biological processes after LamV and WNV infection. (**A**) GO slim terms for up-regulated transcripts. (**B**) GO slim terms for down-regulated transcripts.

The analysis and comparison of mRNA expression profiles of U4.4 cells dual-infected with WNV 24 h post-infection with LamV revealed that 117 (70 up- and 47 down-regulated) and 577 (300 up- and 277 down-regulated) transcripts were significantly DE at 24 h and 48 h post-infection with WNV. Comparing the two transcriptome profiles showed 55 overlapping transcripts between the two time points (Figure 6). Similar to the WNV-only infected cells, many of the up-regulated transcripts were related to immune activity, namely C-type lectin (AALF016234), clip-domain serine protease family B (AALF019859), Defensin (AALF008821), peptidoglycan-recognition protein (AALF020799) and Peptidylprolyl isomerase (AALF012257). Some immune genes were up-regulated at 24 h post-infection with WNV but down-regulated at 48 hpi, such as prophenoloxidase (AALF012716) and clip-domain serine protease family B (AALF020197). Furthermore, among the up-regulated transcripts we observed two signal peptide processing genes, putative microsomal signal peptidase 25 kDa subunit (AALF006247) and signal peptidase complex subunit 3 (AALF003192) (Table 2 and Table S3). The GO enrichment analysis for biological processes resulted in seven and sixteen GO slim terms for the up-regulated transcripts and one and eight GO slim terms for the down-regulated transcript (Figure 7).

### 2.5. Transcripts Differentially Expressed among All Infection Groups

**Figure 8:**
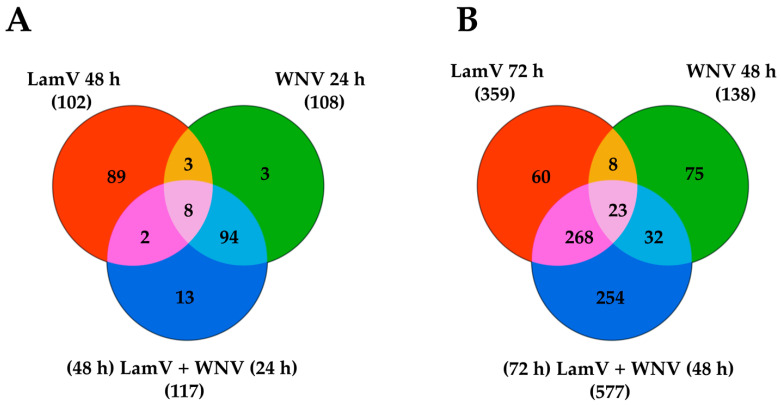
Venn diagram representing the number of differentially expressed transcripts among the different infection groups of U4.4 cells. (**A**) LamV at 48 hpi, WNV at 24 hpi and the dual infection 48 h post-infection with LamV and 24 h post-infection with WNV. (**B**) LamV at 72 hpi, WNV at 48 hpi and the dual infection 72 h post-infection with LamV and 48 h post-infection with WNV.

Comparing the transcriptome profiles among all different infection groups at 24 h post-infection with WNV and 48 h post-infection with LamV reveled eight overlapping transcripts (Figure 8A). Many of the up-regulated transcripts are genes involved in protein processes in the ER, such as BiP/GRP78 (AALF021835), Putative microsomal signal peptidase 25 kDa subunit (AALF006247), Signal peptidase complex subunit 3 (AALF003192) and protein disulfide-isomerase A6 precursor (AALF002466) (Table 3). Interestingly, out of the eight overlapping transcripts, the only down-regulated genes could be observed in LamV-infected U4.4 cells. One of those down-regulated genes most likely encodes for a serine protease (AALF009419) and the other one encodes for the innate immune effector protein prophenoloxidase (AALF012716) (Table 3). Furthermore, the WNV-only and dual-infected U4.4 cells shared 94 DE transcripts at this time point and showed similar fold changes of the up-regulated transcripts common among all infections (Figure 8A and Table 3). The comparison of the same infection groups at 48 h post-infection with WNV and 72 h post-infection with LamV showed that the dual-infected cells were more similar to the LamV-infected cells, with 291 DE transcripts in common (Figure 8B). At these time points, 23 overlapping transcripts among all different infection groups were observed. All these transcripts showed similar up- and down-regulation, except for one transcript coding for the fibrinogen C-terminal domain-containing protein (AALF010275), which was down-regulated in the WNV-infected U4.4 cells but up-regulated in cells infected with LamV or the dual-infection scheme (Table 4). Among the up-regulated genes, the majority was uncharacterized, except for the C-type lectin domain-containing protein (AALF022151), L-lactate dehydrogenase (AALF005588) and Pyrroline-5-carboxylate reductase (AALF003871). The down-regulated genes were mainly involved in DNA replication—DNA helicase (AALF015599, AALF020651 and AALF022735), DNA replication licensing factor MCM7 (AALF006549) and Ribonucleoside-diphosphate reductase (AALF015929 and AALF024232).

## 3. Discussion

Understanding the mosquito–virus interactions and identifying factors important for viral replication in mosquitoes is fundamental in the search for novel arboviruses control strategies. In the present study, we analyzed the altered gene expression upon infection with WNV, the insect-specific LamV and upon a dual-infection scheme, whereby U4.4 cells were pre-infected with LamV 24 h before being challenged with WNV. The qPCR analysis of the supernatant showed that the dual-infected U4.4 cells had a reduced number of WNV RNA copies compared to WNV-only infected cells, which suggest that a prior LamV infection restrain the secondary WNV infection.

When focusing on immune-related genes in WNV-infected U4.4 cells, we observed a cascade of up-regulated immune genes that have previously been described during flavivirus infection of mosquitoes or mosquito-derived cell lines [22,23,24,25,26]. Among the most up-regulated immune-related genes, we observed C-type lysozyme (AALF021807), C-type lectin (AALF016234), defensin (AALF008821), leucine-rich immune protein (AALF016505), Cecropin-A2 (AALF000656), prophenoloxidase (AALF012716), Clip-Domain Serine Protease family B (AALF019859 and AALF020197) and peptidoglycan-Recognition Protein (AALF020799) (Table S2). Furthermore, among the down-regulated transcripts, we observed proteins that belong to the same protein family as the up-regulated immune proteins but with very low similarity to each other, such as C-type lectin (AALF009202), serine protease (AALF013937) and Clip-Domain Serine Protease family D (AALF015014). Many of the immune-related proteins have regulating counterparts in the same protein family and we speculate that these down-regulated transcripts could have a regulatory function. A down-regulation of negative regulators can increase the expression or activity of an effector protein, or contrariwise if up-regulated. Furthermore, the initial immune response was strong, with a high expression of the immune genes at 24 hpi, which was then dampened with a decreased expression at 48 hpi (Table S2). Although we observed this great increase of immune-related genes, it did not seem to halt the WNV infection, with an increasing growth curve over time (Figure 1B). It is hypothesized that the mosquito antiviral immunity limit viral replication, but is unable to effectively clear the virus. This keeps the viral load at a tolerable level and therefore sustaining a persistent infection [13].

LamV-infected U4.4 cells did not induce the same amount of known immune effector proteins as those cells infected with WNV. However, when focusing on 10-fold or higher DE genes in either direction, we observed an up-regulation of different heat-shock proteins (HSPs). These proteins are known to be induced during stress, such as a during a pathogen infection or a heat shock, and act as chaperones to guide misfolded proteins [42]. Among these, we observed endoplasmin (AALF011939), also known as HSP-90, which is connected to the innate immunity of humans [43,44]. We also observed two J-domain-containing proteins, known as HSP-40 (AALF010569 and AALF013640); one BiP/GRP-78 protein, which is an HSP-70 (AALF021835); and two proteins of the GRP-E family (AALF026344 and AALF026694) (Table S1). The HSP-40 and GRP-E proteins both act as co-chaperons for the HSP-70, to increase its activity. Furthermore, we observed two genes within the *lethal(2)-essential-for-life l(2)efl* family (AALF005663 and AALF015016), which encodes HSP-20. The *l(2)efl* family have been shown to be up-regulated upon DENV-2 infection in *Ae. aegypti* mosquitoes [45]. Furthermore, suppression of the *l(2)efl* genes in the CCL-125 cell line showed increased replication of DENV-2, while enhanced expression of the *l(2)efl* genes in the same cell line reduced DENV-2 replication [45]. Among the most down-regulated genes at 72 h post-infection with LamV, we observed two histone H2A (AALF007138 and AALF010649) and three histone H2B genes (AALF010137, AALF014537 and AALF010648) that were down-regulated between 85.16 and 344.4-fold (Table S1). The opposite was observed in a study where the DENV capsid protein was shown to bind the core histone proteins in liver cells and act as a histone mimic, possibly to favor viral replication and repressing the host’s gene transcription; then, liver cells responded to this by initiating an increased production of histones [46]. In summary, the insect-specific LamV did not trigger as prominent immune response as WNV. However, the active viral infection induced a strong stress response in the cell with an increased expression of different HSPs.

U4.4 cells pre-infected with LamV and then challenged with WNV showed the highest amount of DE transcripts among all infection groups. The transcriptome profile of these cells was a mix of genes triggered by both LamV and WNV. Interestingly, although these cells were pre-infected with LamV 24 h before being challenged with WNV, their transcriptome profile was the most similar to the one of WNV-only infected cells at 24 hpi. The same immune effector proteins were expressed at similar levels to those of WNV-only infected cells (Table S3). However, at 48 h post-challenge with WNV, we observed a shift, with a transcriptome profile similar to the one of LamV-infected U4.4 cells at 72 hpi, with an up-regulation of HSPs and down-regulation of the Histone H2A and H2B (Table S3). Furthermore, at this time point, we observed 254 DE transcripts not overlapping with any of the other infections (Figure 8B). Among these transcripts, the most abundant genes observed were two Mitogen-activated protein kinases (MAPK) (AALF018203 and AALF011886). The MAPK is a serine-threonine protein kinase that regulates a broad spectrum of cellular processes, such as growth, metabolism, apoptosis and innate immune responses; however, the understanding of the MAPK signaling cascades in mosquitoes is limited [47]. Unfortunately, for a large proportion of the DE transcripts, we were unable to infer a functional role, due to a lack of annotation in the database. This was especially problematic for the dual-infected cells, where many of the most up- or down-regulated transcripts are currently uncharacterized. However, with such large amount of DE genes in the dual-infected cells, we can assume that the cell viability is decreased.

The RNAi is considered the main antiviral immune response in mosquitoes; however, we did not observe any significant altered gene expression of the main genes involved in the RNAi pathways such as PIWI, Dicer or Ago. This is in line with existing transcriptomics studies performed on viral infections of mosquitoes or mosquito-derived cell lines [22,23,24,25,26]. Surprisingly, these genes seem to have a stable expression during viral infection.

Interestingly, among the genes in common to all infections, we discovered that many of the up-regulated genes are associated with ER functions, including a group of ER-associated signal peptidase complex (SPC) proteins, such as the SPC 25 kDa subunit (AALF006247), the SPC subunit 3 (AALF003192) and the SPC subunit SEC11 (AALF019423). The SPC proteins have shown to be important for proper cleavage of the flavivirus structural proteins (PrM and E) and secretion of viral particles [48]. The silencing of either SPC-1, 2 or 3 in *Drosophila* DL1 cells reduced the infection of WNV and DENV-2 without affecting cell viability; furthermore, gene silencing of SPC-2 in the *Ae. aegypti* cell line Aag2, also reduced WNV infection [48]. The up-regulated SPC genes observed in this study could potentially be targets of choice for gene modification in mosquitoes to reduce transmission of flaviviruses. Other potential targets for modification are the up-regulated HSPs observed in LamV- and dual-infected U4.4 cells. This study provides an overview of the transcriptional response to acute infection of LamV, WNV and the dual-infection scheme in cell culture. Further in vitro and in vivo studies are needed to investigate the effects on the transmission of WNV, of either a prior LamV infection or of the gene modification targets brought up in this discussion.

## 4. Materials and Methods

### 4.1. Cell Culture

*Ae. albopictus* U4.4 mosquito cells (kindly provided by Associate Professor G. Pijlman, Wageningen University) and C6/36 cells (Sigma-Aldrich, Darmstadt, Germany) were cultured at 28 °C without CO_2_ in Leibovitz L-15 medium (Gibco, Paisley, UK) supplemented with 10% fetal bovine serum (FBS; Gibco, Paisley, UK), 10% tryptose phosphate broth (TPB; Gibco, Paisley, UK), Amphoterecin (250 μg/mL; Gibco, Grand Island, NY, USA) and Pen Strep (penicillin 100 U/mL and streptomycin 100 μg/mL; Gibco, Grand Island, NY, USA). Vero E6 cells were cultured at 37 °C with 5% CO_2_ in Dulbecco’s modified Eagle medium (Gibco, Paisley, UK) supplemented with 10% FBS, Pen Strep and 2 mM L-Glutamine (Gibco, Paisley, UK).

### 4.2. Virus Stocks and Virus Titration

The ISFV LamV (2009/FI/Original) was obtained from the European Virus Archive—Global (EVAg). Virus stocks were propagated in C6/36 cells until clear cytopathic effect (CPE) was observed (4 dpi); then, the supernatant was collected, centrifuged and frozen at −80 °C. LamV stock titer could not be obtained with traditional methods. Thus, to quantify the LamV stocks, plasmid standards containing the PCR target region of the virus were ordered (GeneScript Biotech, Leiden, The Netherlands). The plasmids were used to construct a qPCR standard curve and a stock concentration was calculated as RNA copies/mL. RNA extraction and qRT-PCR protocols are described below. WNV (lineage 1) was kindly provided by Professor Åke Lundkvist (Department of Medical Biochemistry and Microbiology/Zoonosis Science Center, Uppsala University, Uppsala, Sweden). WNV stock was grown in Vero E6 cells and the supernatant was collected when clear CPE (3 dpi) was observed. The titer of the WNV stock was determined by a plaque assay, as described in [49].

### 4.3. In Vitro Infection

**Figure 9:**
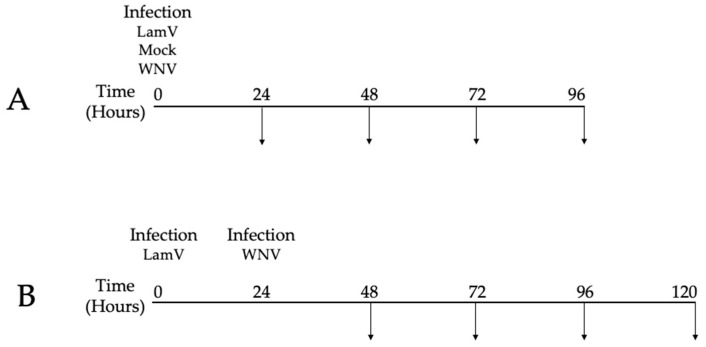
Schematic representation of the experiment schedule. (**A**) Time scheme for single infections. (**B**) Time scheme for cells pre-infected with LamV and challenged with WNV. Arrows represent sampling of cells and supernatant. Cells were only collected between the timepoints 24 to 72 hpi.

On the day of infection, 24-well plates with U4.4 cells at a confluency of 85–90% (approximately 350,000 cells per well) were used. Each infection and timepoint were performed in triplicate. For LamV infections, we added 175,000 RNA copies per well, mixed with 200 μL infection medium (Leibovitz L-15 medium containing 2% FBS and 10% TPB). For WNV infections, the cells were infected at a MOI of 0.1 in 200 μL of infection medium. After one hour of incubation, the inoculum was discarded and 500 μL of Leibovitz L-15 medium supplemented with 5% FBS, 10% TPB and Pen Strep was added. Mock-infected cells were used as control. The supernatant and cells were sampled every 24 h post-infection (hpi) for qPCR and sequencing (Figure 9). All experiments were performed in the BSL-3 facility at the Zoonosis Science Center, Uppsala University, Uppsala, Sweden.

### 4.4. Quantitative PCR

First-strand cDNA was generated using the SuperScript™ III Reverse Transcription kit (Thermo Fisher Scientific, Carlsbad, CA, USA) with Random Hexamers (Thermo Fisher Scientific, Carlsbad, CA, USA), following the manufacturer’s instructions with an input of 5 μL of RNA in a total volume of 20 μL. The qPCR was performed using the iTaq Universal SYBR^®^ Green supermix (Bio-Rad laboratories Inc, Hercules, CA, USA) with 2 μL of template cDNA and 0.5 μM each corresponding virus primer (Table 1) in a total volume of 20 μL per reaction. The qPCR was carried out using the Bio-Rad^®^ CFX96 real-time PCR system (Bio-Rad laboratories Inc, Hercules, CA, USA) with amplification conditions consisting of an initial denaturation at 95 °C for 30 s, followed by 40 cycles of denaturation at 95 °C for 7 s and annealing/extension and plate read at 60 °C 30 s. A melt curve was generated starting at 60 °C with a 0.5 °C increase up to 96 °C.

Primer pairs for the qPCR were designed using the software primer 3 [50] to generate a product between 170 and 200 bp long and Tm of 60 °C. Virus reference genomes were obtained from the NCBI database and are listed, together with the primer sequences, in Table 5. The statistical significance differences were calculated using Student’s *t*-test.

### 4.5. RNA Extractions

RNA used for growth curves and virus titration was extracted from 200 μL of supernatant/virus stocks in 750 μL of TRIzol™ (Thermo Fisher Scientific, Carlsbad, CA, USA). The aqueous phase obtained after the addition of chloroform and the subsequent centrifugation step was collected and diluted (1:1) with freshly prepared 70% ethanol and purified with GeneJet spin columns (Thermo Fisher Scientific, Vilnius, Lithuania). RNA was eluted in 40 μL of nuclease free water and stored at −80 °C until further processed.

RNA used for high-throughput sequencing was isolated from cells that were collected by adding 750 μL of TRIzol™ to the respective wells. The aqueous phase was obtained in the same manner as described above and the RNA was further purified using the mirVana™ PARIS™ Kit (Thermo Fisher Scientific, Vilnius, Lithuania), according to the protocol to isolate large RNA (>200 nt) provided by the manufacturer.

### 4.6. Sequencing of mRNA

Large RNA isolated from infected U4.4 cells was quantified and quality-controlled with the 4150 TapeStation System using the RNA ScreenTape Analysis kit (Agilent Technologies, Santa Clara, CA, USA). Triplicates for each infection and timepoint were submitted to SciLifeLab for library preparation using the Illumina Truseq Stranded mRNA (poly-A selection) kit (San Diego, CA, USA). The libraries were sequenced on an Illumina NovaSeq 6000 sequencer using one lane of a S4 flow cell to a depth of 30–40 million reads per sample with a read length of 2 × 151 bp.

### 4.7. RNA-Seq Data Analysis

The raw transcriptomic FASTQ files were analyzed with bioinformatic Nextflow pipeline nf-core/rnaseq (v3.0) [51]. The pipeline included quality control (FastQC v0.11.9) [52], adapter and quality trimming (Trim Galore! V0.6.6) [53], removal of ribosomal RNA (SortMeRNA v4.2.0) [54] and pseudo-alignment and quantification (Salmon v1.4.0) [55], using the reference *Ae. albopictus* Foshan genome sequence and annotation (version Aalo1.2) retrieved from Vectorbase [56]. The resulting Salmon quant.sf files were further analyzed with an inhouse script in R (doi:10.5281/zenodo.5786479). This script includes a quality control of the data, normalization and DESeq2 analysis. Every infection group was compared with mock-infected cells at the corresponding time point, using the parameters of *p*-adjusted value of ≤0.01 and log_2_ FC of ≥0.5 as cut-offs, following the recommendations found in reference [57]. Lists of significant differentially expressed (DE) transcripts were further analyzed and transcripts IDs were transferred to VectorBase [56] for gene description and Gene Ontology (GO) enrichment analysis. The latter was focused on the sole biological process aspect.

### 4.8. Data Availability

All sequencing data were made publicly available at the NCBI Sequence Read Archive (SRA) under accession number Bioproject ID PRJNA786637.

## 5. Conclusions

This study provides an overview of the transcriptional response to acute infection of WNV, the insect-specific LamV and a dual-infection scheme in an *Ae. albopictus* cell line. The transcriptome profiles of the different infection groups showed a variety of genes with altered expression. WNV-infected cells had an up-regulation of a broad range of immune-related genes, while in LamV-infected cells, many genes related to stress, such as different HSPs, were up-regulated. The transcriptome profile of the dual-infected cells was a mix of up- and down-regulated genes triggered by both viruses, which was more similar to the one of WNV-infected cells in the early time point but shifted towards the one of LamV-infected cells in the later time point.

Since a prior LamV infection seemed to restrain the secondary WNV infection, one of the goals of this study was to find potential gene targets for modification in mosquitoes to reduce transmission of flaviviruses. Interestingly, we observed an up-regulation of SPC proteins in all infection groups. These SPC proteins have shown importance for flavivirus assembly and secretion and could be potential targets for gene modification. Other potential targets for gene modification could be the HSP *l(2)efl* observed in the LamV and dual-infected cells. Further knockdown or overexpression experiments in mosquito-derived cell lines and mosquitoes are needed to study the potential effects on the host and virus transmission.

## Figures and Tables

**Table 1:** List of *Ae. albopictus* overlapping differentially expressed transcripts following WNV infection. Functional categories are based on GO terms for biological processes. The cut-offs used were *p*-adjusted value of ≤0.01 and log_2_ FC of ≥0.5.

Transcript ID	Functional Categories	Gene Description	Fold Change 24 hpi	Fold Change 48 hpi
AALF016234	Immune response	C-type lectin	82.89	17.75
AALF013298		Unspecified product	69.1	14.16
AALF003774	Immune response	Fibrinogen and fibronectin	52.23	18.48
AALF019859	Immune response	Clip-Domain Serine Protease family B	49.51	9.26
AALF008821	Immune response	Defensin antimicrobial peptide	45.85	57.72
AALF026731		Unspecified product	22.22	98.88
AALF008229		Unspecified product	19.82	7.04
AALF025212	Ion homeostasis	Transferrin	15.95	20.69
AALF020799	Immune response	Peptidoglycan-Recognition Protein	14.48	10.23
AALF001195		Unspecified product	12.68	9.79
AALF008452		Unspecified product	12.07	10.83
AALF002418	Metabolic process	Imaginal disc growth factor	10.45	10.37
AALF019963		Unspecified product	9.01	4.88
AALF009200		Unspecified product	8.65	5.95
AALF005588	Metabolic process	L-lactate dehydrogenase	8.11	7.45
AALF004727	Metabolic process	Lipase	7.49	5.5
AALF012590		Unspecified product	7.38	4.56
AALF015711	DNA replication	DNA helicase	6.43	−12.12
AALF022735	DNA replication	DNA helicase	5.48	−13.63
AALF009419		Unspecified product	5.14	4.47
AALF028505		Unspecified product	−13.71	−12.83
AALF008879	Metabolic process	Type IV inositol 5-phosphatase	−16.64	−10.21
AALF015015		Unspecified product	−230.7	22.72

**Table 2:** List of *Ae. albopictus* overlapping differentially expressed transcripts following LamV infection and WNV challenge. Functional categories are based on GO terms for biological processes. Fold changes are shown in hours post-challenge with WNV. The cut-offs used were *p*-adjusted value of ≤0.01 and log_2_ FC of ≥0.5.

Transcript ID	Functional Categories	Gene Description	Fold Change 24 hpi	Fold Change 48 hpi
AALF016234	Immune response	C-type lectin	82.76	22.12
AALF013298		Unspecified product	69.07	23.19
AALF003774	Immune response	Fibrinogen and fibronectin	52.19	9.14
AALF019859	Immune response	Clip-Domain Serine Protease family B	49.47	7.65
AALF008821	Immune response	Defensin antimicrobial peptide	45.81	16.18
AALF026731		Unspecified product	22.2	12.95
AALF020197	Immune response	Clip-Domain Serine Protease family B	15.24	−6.49
AALF020799	Immune response	Peptidoglycan-Recognition Protein	14.47	8.03
AALF007525		Unspecified product	12.61	23.73
AALF016365	Granules fusion	Munc13-4	10.03	6.05
AALF004571		Unspecified product	9.85	122.64
AALF012716	Immune response	Prophenoloxidase	9.77	−5.54
AALF006247	Signal peptide processing	Putative microsomal signal peptidase 25 kDa subunit	8.72	20.65
AALF021835	Response to stress	BiP/GRP78	8.37	31.7
AALF005588	Metabolic process	L-lactate dehydrogenase	8.1	28.92
AALF019952		Unspecified product	7.65	6.1
AALF003990	Metabolic process	Mannosyltransferase	7.13	88.65
AALF027716	Metabolic process	Cytochrome P450	6.64	9.16
AALF003192	Signal peptide processing	Signal peptidase complex subunit 3	6.58	20.26
AALF012257		Peptidylprolyl isomerase	6.55	7.31
AALF019397		Putative reticulocalbin calumenin DNA supercoiling factor	5.97	−4.99
AALF022020	Response to stress	Chaperonin-60kD	5.38	6.94
AALF002466	Response to stress	Protein disulfide-isomerase A6 precursor	5.2	11.04
AALF011939	Response to stress	Endoplasmin	5.12	12.98
AALF002534	Response to stress	Putative heat-shock protein	5.08	8.67
AALF020666	Protein degradation	Ubiquitin	−4.79	−4.99
AALF026911		GPCR Orphan/Putative Class B Family	−7.02	−25.17
AALF016818		Unspecified product	−8.1	−5.85
AALF025231		Unspecified product	−9.13	−20.68
AALF028121		Unspecified product	−9.15	−7.13
AALF015500	DNA/RNA binding	Zinc finger protein	−9.41	−7.58
AALF008709	Transport	Mfs transporter	−10.8	−7.4
AALF011706		Unspecified product	−10.79	−26.44
AALF028505		Unspecified product	−13.72	−65.33
AALF020168		Unspecified product	−13.93	−22.6
AALF008099		Endothelin-converting enzyme	−15.39	−66.8
AALF000742		Unspecified product	−15.81	−54.52
AALF008879	Metabolic process	Type IV inositol 5-phosphatase	−16.65	−60.51
AALF004114		No-mechanoreceptor potential a	−24.69	−45.32
AALF011390		Putative ecdysone-induced protein	−26.61	−21.06
AALF020798		Unspecified product	−29.45	−10.58
AALF009909		Unspecified product	−32.33	−140.61
AALF001259		Unspecified product	−36.13	−36.84
AALF012770	Metabolic process	Aldehyde oxidase	−37.85	−39.12
AALF001105		Unspecified product	−40.91	−26.97
AALF002857		Unspecified product	−42.81	−133.32
AALF002636		Unspecified product	−43.27	−11.27
AALF025810		Unspecified product	−51.86	−28.39
AALF013937	Immune-related	Serine protease	−52.04	−60.31
AALF014375		Unspecified product	−57.5	−72.13
AALF013936		Unspecified product	−78.5	−156.98
AALF006472		Unspecified product	−86.08	−193.34
AALF015014	Immune-related	Clip-Domain Serine Protease family D	−245.49	−75.47
AALF016295		Unspecified product	−249.88	−269.15
AALF014395		Unspecified product	−605.75	−300.78

**Table 3:** List of *Ae. albopictus* overlapping differentially expressed transcripts among LamV at 48 hpi, WNV at 24 hpi and dual-infected cells 24 h post-challenge with WNV. Functional categories are based on GO terms for biological processes. The cut-offs used were *p*-adjusted value of ≤0.01 and log_2_ FC of ≥0.5.

Transcript ID	Functional Categories	Gene Description	Fold Change 48 hpi with LamV	Fold Change 24 hpi with WNV	Fold Change Dual Infection 24 hpi with WNV
AALF004571		DUF3456 domain-containing protein	55.17	9.85	9.86
AALF021835	Response to stress	BiP/GRP78	21.95	8.37	8.37
AALF006247	Signal peptide processing	Putative microsomal signal peptidase 25 kDa subunit	19.29	8.73	8.72
AALF003192	Signal peptide processing	Signal peptidase complex subunit 3	15.78	6.58	6.58
AALF011939	Response to stress	Endoplasmin	11.87	5.12	5.12
AALF002466	Response to stress	protein disulfide-isomerase A6 precursor	8.33	5.2	5.2
AALF009419		Unspecified product	−4.9	5.14	5.13
AALF012716	Immune response	Prophenoloxidase	−12.04	9.78	9.77

**Table 4:** List of *Ae. albopictus* overlapping differentially expressed transcripts among LamV at 72 hpi, WNV 48 hpi and dual-infected cells 48 h post-challenge with WNV. Functional categories are based on GO terms for biological processes. The cut-offs used were *p*-adjusted value of ≤0.01 and log_2_ FC of ≥0.5.

Transcript ID	Functional Categories	Gene Description	Fold Change 72 hpi with LamV	Fold Change 48 hpi with WNV	Fold Change Dual Infection 48 hpi with WNV
AALF010887		Unspecified product	9.29	23.96	13.02
AALF020693		Unspecified product	27.96	16.03	40.21
AALF019136		Unspecified product	6.81	14.5	10.28
AALF014826		Unspecified product	12.18	11.12	22.82
AALF007397		Unspecified product	8.27	7.88	9.94
AALF022151	Immune response	C-type lectin domain-containing protein	7.5	7.47	7.91
AALF005588	Metabolic process	L-lactate dehydrogenase	8.74	7.45	28.92
AALF017030		Unspecified product	9.79	6.86	14.3
AALF007472		Unspecified product	7.8	6.68	13.9
AALF004337		Unspecified product	5.06	5.93	6.77
AALF003871	Metabolic process	Pyrroline-5-carboxylate reductase	14.48	5.21	24.79
AALF010275		Fibrinogen C-terminal domain-containing protein	−6.44	5.05	−6.27
AALF015929	DNA replication	Ribonucleoside-diphosphate reductase	−7.38	−6.52	−9.17
AALF024232	DNA replication	Ribonucleoside-diphosphate reductase	−7.4	−7.42	−9.81
AALF015599	DNA replication	DNA helicase	−6.85	−10.3	−8.42
AALF020651	DNA replication	DNA helicase	−5.72	−10.51	−8.23
AALF000130		dNK domain-containing protein	−12.04	−12.54	−16.06
AALF019880	Oxidationreduction process	Dihydropyrimidine dehydrogenase NADP(+)	−14.35	−12.85	−35.91
AALF013610	Oxidationreduction process	Dihydropyrimidine dehydrogenase NADP(+)	−16.22	−13.14	−42.04
AALF022735	DNA replication	DNA helicase	−5.24	−13.63	−8.08
AALF013129	Metabolic process	Trehalose-6-phosphate synthase	−15.42	−14.88	−23.66
AALF006549	DNA replication	DNA replication licensing factor MCM7	−6.56	−15.21	−9.89
AALF000129	Reverse transcription	Reverse transcriptase domain-containing protein	−21.58	−20	−15.36

**Table 5:** Primer pairs used for qPCR analysis.

Primers	Binding Site	Sequence (5′ → 3′)	Ref
qLamV-F	4659–4678	TGGGTGTTACCGGGTTATGT	FJ606789
qLamV-R	4845–4864	ACGTTCCATTCAGTTTCCAT	
qWNV-F	10490–10508	GAAGTCAGGCCGGAAAGTT	AF260968
qWNV-R	10668–10689	TCTCCGCAGAGTGGCACGCC	

## Data Availability

All sequencing data were made publicly available at the NCBI Sequence Read Archive (SRA) under accession number Bioproject ID PRJNA786637.

## Supplementary Materials

The following are available online at https://www.mdpi.com/article/10.3390/ijms23020875/s1.
