# Supplementary material for: Transcriptome Analysis of an Aedes albopictus Cell Line Single- and Dual-Infected with Lammi Virus and WNV
Source: Int J Mol Sci. 2022 Jan 14;23(2):875. doi: 10.3390/ijms23020875 (PMC8777793; doi:10.3390/ijms23020875)
Supplement: Supplementary file 1 [file ijms-23-00875-s001.zip › Differentially expressed transcripts (_10) after dual-infection.pdf]

Supplementary Table S3. List of *Ae. albopictus* differentially expressed transcripts (>10 fold change) post dual-infection. Fold change is shown post WNV challenge. Bold numbers have p-adjust value ≤0.01

Transcript ID	Gene description	Fold change 24 hpi with WNV	Fold change 48 hpi with WNV
AALF021807	C-Type Lysozyme (Lys-E)	**433,67**	5,25
AALF003252	Unspecified product	**141,8**	6,88
AALF016234	C-Type Lectin	**82,76**	**22,12**
AALF010414	Beta-hexosaminidase b	**73,13**	1,29
AALF013298	Unspecified product	**69,07**	**23,19**
AALF022426	Unspecified product	**58,18**	1,24
AALF009966	Unspecified product	**57,49**	3,8
AALF003774	Fibrinogen and fibronectin	**52,19**	**9,14**
AALF019859	Clip-Domain Serine Protease family B	**49,47**	**7,65**
AALF008821	Defensin anti-microbial peptide	**45,81**	**16,18**
AALF008776	Aromatic amino acid decarboxylase	**44,81**	2,13
AALF022425	Unspecified product	**30,37**	3,47
AALF009414	Putative secreted protein	**27,46**	4,81
AALF026731	Unspecified product	**22,2**	**12,95**
AALF001194	Unspecified product	**21,11**	-1,02
AALF008229	Unspecified product	**19,8**	1,13
AALF001093	Calreticulin	**18,74**	1,48
AALF007742	Unspecified product	**18,38**	-4,01
AALF020341	Unspecified product	**18,34**	-1,1
AALF023135	Unspecified product	**18,05**	-1,29
AALF016505	Leucine-rich immune protein	**16,82**	-1,43
AALF025212	Transferrin	**15,93**	-1,08
AALF020197	Clip-Domain Serine Protease family B	**15,24**	**-6,49**
AALF020799	Peptidoglycan Recognition Protein	**14,47**	**8,03**
AALF000812	Unspecified product	**14,35**	-2,81
AALF001195	Unspecified product	**12,67**	1,22
AALF007525	Unspecified product	**12,61**	**23,73**
AALF008452	Unspecified product	**12,06**	1,2
AALF015567	Cystathionine beta-synthase	**11,47**	2,07
AALF014689	Fibrinogen and fibronectin	**10,7**	-2,12
AALF002418	Imaginal disc growth factor	**10,43**	3,14
AALF023540	Unspecified product	**10,41**	1
AALF023089	Serine--pyruvate aminotransferase	**10,31**	2,58
AALF001232	Pyruvate carboxylase	**10,23**	-1,45
AALF016365	Munc13-4	**10,03**	**6,05**
AALF011706	Unspecified product	**-10,79**	**-26,44**
AALF008709	Mfs transporter	**-10,8**	**-7,4**
AALF028505	Unspecified product	**-13,72**	**-65,33**
AALF020168	Unspecified product	**-13,93**	**-22,6**

AALF025541	Unspecified product	**-15,32**	-7,35
AALF008099	Endothelin-converting enzyme	**-15,39**	**-66,8**
AALF000742	Unspecified product	**-15,81**	**-54,52**
AALF010788	Unspecified product	**-16,19**	-3,04
AALF008879	Type IV inositol 5-phosphatase	**-16,65**	**-60,51**
AALF016756	Sugar transporter	**-16,65**	-3,67
AALF005838	Unspecified product	**-19,31**	-3,52
AALF022022	cdkl1/4	**-23,59**	-3,78
AALF004114	No-mechanoreceptor potential a	**-24,69**	**-45,32**
AALF011390	Putative ecdysone-induced protein	**-26,61**	**-21,06**
AALF020798	Unspecified product	**-29,45**	**-10,58**
AALF000142	Unspecified product	**-31,21**	-9,07
AALF009909	Unspecified product	**-32,33**	**-140,61**
AALF001259	Unspecified product	**-36,13**	**-36,84**
AALF014478	Heat shock protein HSP70	**-37,21**	-2,23
AALF012770	Aldehyde oxidase	**-37,85**	**-39,12**
AALF001105	Unspecified product	**-40,91**	**-26,97**
AALF002857	Unspecified product	**-42,81**	**-133,32**
AALF002636	Unspecified product	**-43,27**	**-11,27**
AALF014479	Unspecified product	**-45,78**	-4,49
AALF025810	Unspecified product	**-51,86**	**-28,39**
AALF013937	Serine protease	**-52,04**	**-60,31**
AALF014375	Unspecified product	**-57,5**	**-72,13**
AALF014481	Unspecified product	**-61,13**	-4,53
AALF027745	Microtubule-associated protein	**-61,99**	-12,41
AALF009202	C-Type Lectin (CTL)	**-65,3**	-13,8
AALF013936	Unspecified product	**-78,5**	**-156,98**
AALF006472	Unspecified product	**-86,08**	**-193,34**
AALF018782	Unspecified product	**-140,73**	-7,07
AALF015015	Unspecified product	**-230,79**	-2,78
AALF015014	Clip-Domain Serine Protease family D	**-245,49**	**-75,47**
AALF016295	Unspecified product	**-249,88**	**-269,15**
AALF014395	Unspecified product	**-605,75**	**-300,78**
AALF028496	Unspecified product	**-78146,04**	-2,52
AALF005731	Zinc finger protein	**-108806,97**	-3,06
AALF010569	Unspecified product	55,04	**6440,46**
AALF001962	Unspecified product	-11,56	**609,49**
AALF013640	Unspecified product	2,62	**174,41**
AALF018203	Mitogen-activated protein kinase	1,67	**146,9**
AALF004571	Unspecified product	**9,85**	**122,64**
AALF013285	Unspecified product	-1,1	**106,95**
AALF019759	Unspecified product	1,81	**88,79**
AALF003990	Mannosyltransferase	**7,13**	**88,65**

AALF003128	4-nitrophenylphosphatase	7,55	**66,68**
AALF003110	Unspecified product	2,94	**57,86**
AALF005738	Unspecified product	3,28	**52,57**
AALF013715	Unspecified product	5,78	**44,94**
AALF025486	Unspecified product	4,16	**44,72**
AALF011149	Unspecified product	1,37	**44,14**
AALF011886	Mitogen-activated protein kinase	3,5	**43,42**
AALF010775	Unspecified product	42,41	**43,12**
AALF011414	Unspecified product	2,71	**40,53**
AALF020693	Unspecified product	-2,54	**40,21**
AALF022006	Unspecified product	14,55	**39,8**
AALF003569	Unspecified product	9,47	**39,52**
AALF002752	Unspecified product	3,56	**37,95**
AALF001937	Unspecified product	4,45	**37,7**
AALF021815	Sulfhydryl oxidase	1,83	**33,37**
AALF027170	Unspecified product	-1,18	**32,06**
AALF026344	Unspecified product	2,55	**31,71**
AALF021835	BiP/GRP78	**8,37**	**31,7**
AALF027168	Pcdc2/rp-8	6,72	**29,28**
AALF005588	L-lactate dehydrogenase	**8,1**	**28,92**
AALF026694	GrpE protein homolog	4,77	**28,84**
AALF023086	Sil1	1,5	**28,59**
AALF012753	Unspecified product	12,07	**28,33**
AALF024273	39S ribosomal protein L34	2,68	**26,79**
AALF019596	Unspecified product	2,78	**26,78**
AALF007470	Unspecified product	1,58	**25,27**
AALF003871	Unspecified product	2,78	**24,79**
AALF005644	Protein KRTCAP2 homolog	-1,05	**23,85**
AALF017272	60S ribosome subunit biogenesis protein NIP7 homolog	2,63	**23,72**
AALF028423	Poly [ADP-ribose] polymerase	1,11	**23,69**
AALF019485	Unspecified product	3,6	**23,68**
AALF007387	Unspecified product	3,27	**23,54**
AALF003976	Unspecified product	10,42	**23,4**
AALF014826	Unspecified product	1,78	**22,82**
AALF006960	Derlin	2,33	**21,84**
AALF014725	Unspecified product	5,5	**21,4**
AALF008938	Putative vesicle coat complex copi zeta subunit	2,98	**21,29**
AALF006247	Putative microsomal signal peptidase 25 kDa subunit	**8,72**	**20,65**
AALF003192	Signal peptidase complex subunit 3	**6,58**	**20,26**
AALF019423	Signal peptidase complex catalytic subunit SEC11	5,27	**19,83**
AALF026705	Putative mitochondrial import inner membrane translocase subunit tim8	1,19	**19,77**
AALF005816	ER membrane protein complex subunit 4	3,83	**18,61**

AALF018519	Unspecified product	2,37	**18,5**
AALF017894	Pyrroline-5-carboxylate reductase	1,73	**18,46**
AALF010277	L-lactate dehydrogenase	4,25	**18,42**
AALF017936	Unspecified product	1,21	**18,35**
AALF027267	39S ribosomal protein L22	2,55	**18,28**
AALF019139	Hect E3 ubiquitin ligase	1,24	**17,75**
AALF021211	Unspecified product	4,56	**16,31**
AALF018192	Kinase	3,69	**16,17**
AALF006811	Metaxin	6,87	**16,14**
AALF023408	Unspecified product	2,68	**16,02**
AALF013670	Unspecified product	6,32	**15,93**
AALF017066	Unspecified product	1,6	**15,88**
AALF002862	39S mitochondrial ribosomal protein L28	3,2	**15,52**
AALF028288	Unspecified product	1,57	**15,45**
AALF016188	Eukaryotic translation initiation factor	5,44	**15,34**
AALF012726	Unspecified product	2,67	**14,99**
AALF015083	Unspecified product	2,3	**14,81**
AALF023527	Unspecified product	1,63	**14,76**
AALF025878	Unspecified product	2,99	**14,65**
AALF002306	Unspecified product	5,12	**14,64**
AALF002436	Unspecified product	1,83	**14,64**
AALF016399	Unspecified product	3,82	**14,59**
AALF015963	Unspecified product	-1,04	**14,48**
AALF017030	Unspecified product	3,04	**14,3**
AALF010131	Unspecified product	4,79	**14,26**
AALF017935	Stearoyl-coa desaturase	-1,07	**14**
AALF024308	Unspecified product	2,94	**13,99**
AALF007472	Unspecified product	1,48	**13,9**
AALF003428	Unspecified product	10,77	**13,85**
AALF027123	Long-chain-fatty-acid coa ligase	3,29	**13,77**
AALF001336	Putative selenoprotein g	3,02	**13,71**
AALF023156	tRNA (adenine(58)-N(1))-methyltransferase catalytic subunit TRMT61A	2,66	**13,46**
AALF002987	Unspecified product	1,52	**13,31**
AALF008836	Unspecified product	1,58	**13,26**
AALF017325	Unspecified product	1,27	**13,12**
AALF020872	Putative secreted protein	2,34	**13,05**
AALF021933	Unspecified product	1,31	**13,04**
AALF010887	Unspecified product	-1,08	**13,02**
AALF008678	Choline-phosphate cytidylyltransferase a, b	9,83	**12,99**
AALF011939	Endoplasmin	**5,12**	**12,98**
AALF021504	Unspecified product	2,43	**12,79**
AALF015904	Unspecified product	3,51	**12,77**

AALF026342	Unspecified product	3,08	**12,76**
AALF001505	Cell adhesion molecule	1,27	**12,75**
AALF006812	Short-chain dehydrogenase	2,02	**12,34**
AALF010373	Phosphoglycerate mutase	1,36	**12,34**
AALF008913	Unspecified product	2,7	**12,15**
AALF006680	Unspecified product	2,47	**12,03**
AALF020200	Mitochondrial ornithine transporter	3,24	**12,01**
AALF009639	Unspecified product	6,06	**11,84**
AALF012016	DnaJ homolog subfamily B member 11 precursor	2,05	**11,77**
AALF020282	Unspecified product	3,15	**11,72**
AALF002846	Putative cpij005033 nucleolar protein nhp2	4,43	**11,71**
AALF001439	Unspecified product	1,36	**11,71**
AALF000613	Unspecified product	2,5	**11,65**
AALF014036	Unspecified product	2,26	**11,57**
AALF016160	Activating signal cointegrator 1 complex subunit 3, helc1	1,84	**11,46**
AALF013787	Unspecified product	-1,07	**11,36**
AALF016639	Unspecified product	-1,09	**11,23**
AALF026969	Unspecified product	2,22	**11,07**
AALF002466	Protein disulfide-isomerase A6 precursor	5,2	**11,04**
AALF003447	Unspecified product	1,96	**10,92**
AALF013154	Unspecified product	4,78	**10,9**
AALF019760	Unspecified product	7,48	**10,43**
AALF020008	Putative growth hormone-induced protein	6,29	**10,4**
AALF019136	Unspecified product	-1,68	**10,28**
AALF001501	Phosphoglucomutase	2,24	**10,27**
AALF022744	Mitochondria associated granulocyte macrophage csf signaling molecule	1,04	**10,21**
AALF014037	Cle7	2,48	**10,05**
AALF009537	Unspecified product	-1,03	**10**
AALF003854	Repressor of RNA polymerase III transcription MAF1	-1,16	**-10,05**
AALF023601	Peroxidasin	4,7	**-10,12**
AALF013429	Unspecified product	-6,17	**-10,13**
AALF006047	Unspecified product	-3,13	**-10,17**
AALF026258	DNA helicase	-2,55	**-10,19**
AALF023157	Unspecified product	-1,32	**-10,47**
AALF003039	Prophenoloxidase	-1,52	**-10,48**
AALF015542	Unspecified product	2,05	**-10,57**
AALF008037	Unspecified product	1,07	**-10,65**
AALF016899	Unspecified product	-1,67	**-10,7**
AALF023425	Unspecified product	-2,87	**-10,73**
AALF011299	Gamma glutamyl transpeptidases	-6,92	**-10,92**
AALF017425	Unspecified product	4,66	**-10,93**
AALF021839	Sodium/solute symporter	-3,69	**-11,1**

AALF027385	Adenylate cyclase	-2,16	**-11,11**
AALF005533	Unspecified product	-3,84	**-11,48**
AALF011858	Unspecified product	-8,58	**-11,66**
AALF008809	Nidogen	2,1	**-11,94**
AALF024686	Unspecified product	-3,35	**-11,99**
AALF015269	Unspecified product	-3,8	**-12,26**
AALF020706	Bifunctional purine biosynthesis protein	-1,77	**-12,54**
AALF019525	Unspecified product	-8,92	**-12,58**
AALF000395	Unspecified product	1,42	**-13,67**
AALF018338	F-spondin	1,41	**-13,93**
AALF003944	Unspecified product	-6,28	**-14,29**
AALF025462	Unspecified product	-15,65	**-14,74**
AALF000129	Unspecified product	4,62	**-15,36**
AALF008893	Unspecified product	-2,66	**-15,6**
AALF024829	Brain chitinase and chia	-2,11	**-15,73**
AALF011491	Unspecified product	1,83	**-15,84**
AALF015943	Methylenetetrahydrofolate dehydrogenase	2,08	**-15,87**
AALF008765	Glutamate synthase	-1,18	**-16,02**
AALF000130	Unspecified product	3,55	**-16,06**
AALF022389	Unspecified product	-1,36	**-16,26**
AALF007615	Unspecified product	-9,62	**-16,35**
AALF005637	Unspecified product	-2,92	**-16,39**
AALF007101	Unspecified product	-1,95	**-16,99**
AALF019868	F-spondin	2,69	**-17,25**
AALF007246	Unspecified product	-1,25	**-17,35**
AALF009035	Cyclin a	-4,45	**-17,38**
AALF007570	Clip-Domain Serine Protease family B. Protease homologue	1,65	**-17,51**
AALF009765	Glutamate synthase	-1,77	**-17,84**
AALF027905	Prophenoloxidase	1,78	**-18,05**
AALF023670	Unspecified product	-2,97	**-18,05**
AALF005743	Unspecified product	-4,54	**-18,17**
AALF012739	Alpha-mannosidase	-2,52	**-18,34**
AALF027708	Unspecified product	-2,68	**-18,36**
AALF013089	Unspecified product	-2,8	**-18,83**
AALF021923	Gamma-glutamyl hydrolase	1,27	**-18,85**
AALF012022	Sodium-dependent phosphate transporter	-1,57	**-19,08**
AALF016175	Matrix metalloproteinase	-2,76	**-19,59**
AALF014285	Unspecified product	-8,93	**-19,82**
AALF002486	Unspecified product	-14,81	**-20,11**
AALF006132	Unspecified product	-7,01	**-20,49**
AALF025231	Unspecified product	**-9,13**	**-20,68**
AALF017372	Translin associated factor x	-7,52	**-20,69**
AALF016456	Unspecified product	-12,44	**-21,08**

AALF003366	Unspecified product	-13,7	**-21,34**
AALF027074	Unspecified product	-7,49	**-21,61**
AALF028546	Unspecified product	-6,17	**-21,84**
AALF016286	Unspecified product	-2,81	**-21,87**
AALF012694	Putative apolipoprotein d/lipocalin	-3,92	**-22,44**
AALF022970	Unspecified product	-2	**-22,52**
AALF024663	GPCR Octopamine/Tyramine Family	-21,55	**-22,72**
AALF002599	Unspecified product	-2,44	**-22,85**
AALF011816	Unspecified product	-2	**-23,22**
AALF018011	Unspecified product	-1,62	**-23,59**
AALF013129	Trehalose-6-phosphate synthase	-1,93	**-23,66**
AALF003849	Unspecified product	-6,36	**-24,34**
AALF016501	Unspecified product	-7,73	**-24,6**
AALF016566	Unspecified product	-2,33	**-25,09**
AALF026911	GPCR Orphan/Putative Class B Family	**-7,02**	**-25,17**
AALF020305	Carboxylic ester hydrolase	-5,35	**-25,29**
AALF027964	Unspecified product	-11,8	**-25,36**
AALF023403	Unspecified product	-7,12	**-25,75**
AALF028503	Defense repressor	-5,65	**-26,16**
AALF012970	Unspecified product	1,93	**-26,36**
AALF021548	GPCRG astrin/Bombesin Family	-13,46	**-26,74**
AALF021556	Unspecified product	-26,66	**-28,34**
AALF025810	Unspecified product	**-51,86**	**-28,39**
AALF010876	Unspecified product	-5,21	**-28,97**
AALF008397	Unspecified product	-8,95	**-29,55**
AALF006674	Unspecified product	-11,91	**-29,68**
AALF006581	Unspecified product	1,36	**-29,76**
AALF006673	Unspecified product	-6,18	**-31,67**
AALF010019	Brain chitinase and chia	-3,66	**-33,56**
AALF004309	Putative sodium/potassium-transporting atpase subunit beta-2	4,1	**-34,15**
AALF019880	Dihydropyrimidine dehydrogenase [NADP(+)]	1,25	**-35,91**
AALF010137	Histone H2B	1,34	**-38,11**
AALF014933	Prophenoloxidase	3,43	**-38,44**
AALF020399	Unspecified product	-38,38	**-39,25**
AALF013610	Dihydropyrimidine dehydrogenase [NADP(+)]	1,27	**-42,04**
AALF022513	Unspecified product	-2,36	**-44,29**
AALF016111	Unspecified product	-9,53	**-44,64**
AALF012955	Serine protease	-3,92	**-45,02**
AALF014537	Histone H2B	2,06	**-50,14**
AALF003951	Unspecified product	-7,78	**-53,28**
AALF010648	Histone H2B	1,91	**-54,45**
AALF005415	Unspecified product	-27,49	**-57,41**
AALF020202	Unspecified product	-9,21	**-58,36**

AALF024124	Cytochrome P450	3,79	**-62,99**
AALF004118	Unspecified product	-8,76	**-65,39**
AALF010649	Histone H2A	-1,26	**-86,69**
AALF011637	Unspecified product	-5,25	**-96,12**
AALF012023	Unspecified product	-8,18	**-101,26**
AALF016470	Carboxylic ester hydrolase	-1,19	**-126,43**
AALF019822	Unspecified product	-80,8	**-152,61**
AALF010790	Unspecified product	-2,76	**-168,37**
AALF022750	Unspecified product	-10,19	**-209,59**
AALF019479	Unspecified product	-39,56	**-321,03**
AALF013172	Alpha-amylase	-4,84	**-370,93**
AALF012014	Unspecified product	-19,42	**-566,3**
AALF023401	Unspecified product	-130,49	**-645,54**
AALF018987	Unspecified product	-1,11	**-2938,92**
