# Supplementary material for: Transcriptome Analysis of an Aedes albopictus Cell Line Single- and Dual-Infected with Lammi Virus and WNV
Source: Int J Mol Sci. 2022 Jan 14;23(2):875. doi: 10.3390/ijms23020875 (PMC8777793; doi:10.3390/ijms23020875)
Supplement: Supplementary file 1 [file ijms-23-00875-s001.zip › Differentially expressed transcripts (_10) post-infection with LamV.pdf]

Supplementary Table S1. List of *Ae. albopictus* differentially expressed transcripts (>10 fold change) post-infection with LamV. Bold numbers have p-adjust value ≤0.01.

Transcript ID	Gene description	fold change 24 hpi with LamV	fold change 48 hpi with LamV	Fold change 72 hpi with LamV
AALF020879	Unspecified product	**12,61**	**7,6**	2,44
AALF012914	Unspecified product	**11,92**	**7,94**	3,77
AALF005471	40S ribosomal protein S21	**11,38**	**13,49**	**7,08**
AALF002626	40S ribosomal protein S21	**10,94**	**8**	**8,62**
AALF017826	COX6C domain-containing protein	**10,08**	**12,89**	4,54
AALF005278	Unspecified product	11,77	**241938955**	29,56
AALF010569	J domain-containing protein	26,5	**5787,58**	1229,88
AALF004571	DUF3456 domain-containing protein	2,34	**55,17**	**85,91**
AALF010775	CHCH domain-containing protein	-1,71	**49,97**	**70,98**
AALF003990	Mannosyltransferase	3,09	**34,71**	**42,1**
AALF005663	Lethal(2)essential for life protein	2,76	**23,89**	**17,34**
AALF006960	Derlin	3,09	**22,59**	**14,45**
AALF011414	Unspecified product	4,1	**22,33**	**30,57**
AALF021835	BiP/GRP78	1,13	**21,95**	**18,06**
AALF006247	Putative microsomal signal peptidase 25 kDa subunit	1,69	**19,29**	**17,61**
AALF004828	Putative secreted protein	1,57	**19,05**	6,27
AALF025486	Unspecified product	1,19	**18,49**	**30,07**
AALF013670	Unspecified product	4,23	**18,26**	**13,38**
AALF014036	DUF4536 domain-containing protein	4,54	**18,014**	11,57
AALF002158	Unspecified product	7,68	**17,49**	4,19
AALF005738	Unspecified product	1,73	**16,85**	**30,78**
AALF010933	Unspecified product	3,68	**16,29**	2,56
AALF003192	Signal peptidase complex subunit 3	2,28	**15,78**	**12,19**
AALF021815	Sulfhydryl oxidase	3,06	**14,91**	**26,66**
AALF009959	UDP-galactose transporter	1,91	**14,32**	5,33
AALF002306	Unspecified product	2,44	**14,27**	**10,24**
AALF020693	Unspecified product	4,45	**14,19**	**27,96**
AALF013715	Unspecified product	2,31	**13,07**	**30,66**
AALF026819	Unspecified product	1,77	**12,74**	4,98
AALF002752	Unspecified product	1,33	**12,53**	**19,52**
AALF005644	Protein KRTCAP2 homolog	5,37	**12,39**	**19,59**
AALF017325	Unspecified product	5,09	**11,93**	**12,31**
AALF011939	Endoplasmin	-1,03	**11,87**	**6,12**
AALF023162	Sep15_SelM domain-containing protein	3,78	**11,55**	5,95
AALF012595	EGF-like domain-containing protein	1,7	**10,85**	5,93
AALF002612	Putative mitochondrial respiratory chain complex i	6,11	**10,79**	2,89
AALF005815	Unspecified product	5,73	**10,65**	4,09

AALF012691	Dolichyl-diphosphooligosaccharide--protein glycosyltransferase subunit DAD1	2,75	**10,07**	5,41
AALF021504	Unspecified product	1,14	**10,01**	**6,75**
AALF013722	DNA polymerase	-1,5	**-10,74**	-5,1
AALF012716	Prophenoloxidase	-1,01	**-12,04**	**-14,42**
AALF003040	Unspecified product	-1,2	**-13,45**	-7,45
AALF016470	Carboxylic ester hydrolase	-1,79	**-16,91**	**-87,5**
AALF013640	J domain-containing protein	1,61	28,45	**165,75**
AALF012753	CHCH domain-containing protein	-1,22	12,43	**41,5**
AALF003110	Unspecified product	1,49	**9,49**	**39,45**
AALF003128	4-nitrophenylphosphatase	1,91	13,52	**32,13**
AALF011149	LITAF domain-containing protein	3,96	3,81	**29,8**
AALF007387	Unspecified product	2,03	2,59	**24,95**
AALF022006	Belongs to the HAD-like hydrolase superfamily	1,09	9,24	**23,94**
AALF026344	Belongs to the GrpE family	1,89	4	**22,5**
AALF026694	GrpE protein homolog	1,71	5,85	**22,07**
AALF028423	Poly [ADP-ribose] polymerase	1,81	**8,72**	**20,94**
AALF019485	Brix domain-containing protein	1,99	3,64	**19,67**
AALF024199	HSP-70 binding protein	1,96	8,66	**19,28**
AALF015016	Lethal(2)essential for life protein, l2efl	1,4	**8,95**	**18,32**
AALF019596	Unspecified product	2,23	9,55	**17,42**
AALF023086	sil1	1,94	**8,43**	**16,99**
AALF024273	39S ribosomal protein L34	5,89	4,22	**16,02**
AALF007525	LITAF domain-containing protein	1,21	4,42	**15,98**
AALF027170	Unspecified product	-1,58	1,25	**15,88**
AALF017272	60S ribosome subunit biogenesis protein NIP7 homolog	3,1	1,65	**15,14**
AALF026705	Putative mitochondrial import inner membrane translocase subunit tim8	3,79	**7,09**	**14,53**
AALF008938	Putative vesicle coat complex copi zeta subunit	3,62	**9,19**	**14,5**
AALF003871	Pyrroline-5-carboxylate reductase	1,59	4,59	**14,48**
AALF019391	Unspecified product	2,09	7,57	**14,47**
AALF001937	Unspecified product	-1,14	-1,04	**14,4**
AALF028288	Unspecified product	-1,03	4,61	**13,69**
AALF015083	Unspecified product	1,67	2,97	**13,55**
AALF009537	Unspecified product	4,04	5,38	**13,51**
AALF005816	ER membrane protein complex subunit 4	2,63	**6,19**	**13,48**
AALF020200	Mitochondrial ornithine transporter	1	4,54	**12,63**
AALF017894	Pyrroline-5-carboxylate reductase	2,14	4,63	**12,61**
AALF018519	Nudix hydrolase domain-containing protein	3,38	6,5	**12,58**
AALF027267	39S ribosomal protein L22	3,8	5,8	**12,35**
AALF014826	Unspecified product	1,94	4,75	**12,18**
AALF001439	F-box domain-containing protein	1,17	2,07	**12,08**
AALF016188	Eukaryotic translation initiation factor	1,15	3,63	**12,08**

AALF002862	39S mitochondrial ribosomal protein L28	1,95	3,86	**11,74**
AALF020008	Putative growth hormone-induced protein	-1,02	**5,97**	**11,55**
AALF019423	Signal peptidase complex catalytic subunit SEC11	1,83	**9,96**	**11,53**
AALF001336	Putative selenoprotein g	1,76	**7,66**	**11,45**
AALF017936	Unspecified product	1,01	2,76	**11,22**
AALF016828	Unspecified product	1,91	4,99	**10,96**
AALF022971	RING-CH-type domain-containing protein	-1,12	3,28	**10,7**
AALF023408	Unspecified product	1,85	1,78	**10,51**
AALF001047	Unspecified product	6,11	7,79	**10,24**
AALF000279	PHB domain-containing protein	-1,28	4,25	**10,23**
AALF020872	Putative secreted protein	3,11	1,81	**10,22**
AALF015963	Unspecified product	2,45	3,88	**10,13**
AALF003447	Unspecified product	2,49	4,23	**10,13**
AALF002436	DUF155 domain-containing protein	1,63	2,98	**10,01**
AALF015692	Unspecified product	-1,76	-3,97	**-10,02**
AALF008397	ANK_REP_REGION domain-containing protein	-2,3	-2,38	**-10,1**
AALF028546	GH16 domain-containing protein	1,22	-5,77	**-10,16**
AALF017425	VWFC domain-containing protein	-1,73	-3,33	**-10,56**
AALF013861	Unspecified product	-2,16	-4,53	**-10,75**
AALF006764	Reverse transcriptase domain-containing protein	-2,19	-3,66	**-11,09**
AALF016175	Matrix metalloproteinase	-1,12	-4,22	**-11,11**
AALF008809	Nidogen	-1,14	-3,6	**-11,13**
AALF022970	Unspecified product	-1,43	-1,99	**-11,22**
AALF002486	Reverse transcriptase Ty1/copia-type domain-containing protein	-2,33	-1,96	**-11,23**
AALF025212	Transferrin	-1,06	-2,2	**-11,31**
AALF023670	Unspecified product	1,12	-2,12	**-11,41**
AALF021839	Sodium/solute symporter	-1,11	-2,13	**-11,49**
AALF025224	Integrase catalytic domain-containing protein	1,37	-1,81	**-11,5**
AALF023157	Tyrosinase_Cu-bd domain-containing protein	-1,33	-3,25	**-11,57**
AALF003039	Prophenoloxidase	-1,24	-3,68	**-11,71**
AALF000130	dNK domain-containing protein	1,31	-3,9	**-12,04**
AALF011858	Vitellogenin domain-containing protein	-2,73	-2,63	**-12,19**
AALF017340	RGS domain-containing protein	-1,61	-2,27	**-12,81**
AALF008038	Y-box binding protein	-2,35	-2,77	**-12,98**
AALF005743	Reverse transcriptase Ty1/copia-type domain-containing protein	-1,17	-5,88	**-13,17**
AALF021923	Gamma-glutamyl hydrolase	-1,04	**-6,21**	**-13,49**
AALF008037	Unspecified product	-1,8	-4,48	**-13,61**
AALF018011	Unspecified product	-1,33	-2,11	**-13,68**
AALF007570	Clip-Domain Serine Protease family B	-1,02	-5,2	**-13,82**
AALF004790	Unspecified product	-1,45	-3,6	**-13,94**
AALF012770	Aldehyde oxidase	-1,31	-1,99	**-14,1**

AALF005637	Belongs to the short-chain dehydrogenases/reductases (SDR) family	2,41	-1,64	**-14,23**
AALF019880	Dihydropyrimidine dehydrogenase [NADP(+)]	-1,58	-1,48	**-14,35**
AALF011617	Unspecified product	-1,15	-3,14	**-14,54**
AALF023601	Peroxidasin	-2,29	-4,08	**-14,55**
AALF013089	tRNA_NucTransf2 domain-containing protein	-1,07	-2,31	**-15,4**
AALF013129	Trehalose-6-phosphate synthase	-2	-1,97	**-15,42**
AALF001667	Unspecified product	-1,31	-3,25	**-15,86**
AALF006674	Integrase catalytic domain-containing protein	-1,27	-2,25	**-15,87**
AALF013610	Dihydropyrimidine dehydrogenase [NADP(+)]	-1,94	-1,75	**-16,22**
AALF008099	Endothelin-converting enzyme	-1,48	-2,33	**-16,37**
AALF006673	Retrotrans_gag domain-containing protein	-1,72	-1,77	**-16,56**
AALF016666	FHA domain-containing protein	-1,46	-3,44	**-16,9**
AALF004114	No-mechanoreceptor potential a	-1,23	-4,37	**-17,22**
AALF024124	Cytochrome P450	-1,1	-2,2	**-17,94**
AALF011816	Integrase catalytic domain-containing protein	1,13	-1,55	**-18,07**
AALF007569	Peptidase S1 domain-containing protein	1,53	-3,46	**-19,17**
AALF016566	Unspecified product	-2,79	-3,48	**-19,75**
AALF000129	Reverse transcriptase domain-containing protein	-1,05	-2,82	**-21,58**
AALF019918	Unspecified product	-1,5	-3,47	**-22,73**
AALF016286	B box-type domain-containing protein	-1,35	-4,14	**-23,66**
AALF014933	Prophenoloxidase	-1,12	-3,69	**-29,38**
AALF004309	Putative sodium/potassium-transporting atpase subunit beta-2	-1,05	-3,4	**-31,2**
AALF006581	CHK domain-containing protein	1,13	-2,76	**-32,13**
AALF012955	Serine protease	1,34	-5,56	**-38,1**
AALF022750	Unspecified product	-4,33	-9,64	**-47,74**
AALF020600	RT_RNaseH domain-containing protein	-2,19	-2,62	**-70,65**
AALF007138	Histone H2A	1,56	1,65	**-85,16**
AALF003951	Reverse transcriptase Ty1/copia-type domain-containing protein	-1,13	-2,67	**-104,44**
AALF010649	Histone H2A	4,09	1,37	**-122,87**
AALF010137	Histone H2B	2,31	-2,24	**-149,04**
AALF014537	Histone H2B	1,14	-2,71	**-223,22**
AALF024096	Unspecified product	-1,28	-3,16	**-342,06**
AALF010648	Histone H2B	1,41	-2,11	**-344,4**
AALF028233	Unspecified product	-4,19	2,06	**-988301,15**
