# Supplementary material for: Transcriptome Analysis of an Aedes albopictus Cell Line Single- and Dual-Infected with Lammi Virus and WNV
Source: Int J Mol Sci. 2022 Jan 14;23(2):875. doi: 10.3390/ijms23020875 (PMC8777793; doi:10.3390/ijms23020875)
Supplement: Supplementary file 1 [file ijms-23-00875-s001.zip › Differentially expressed transcripts (_10) post-infection with WNV.pdf]

Supplementary Table S2. List of *Ae. albopictus* differentially expressed transcripts (>10 fold change) post-infection with WNV. Bold numbers have p-adjust value ≤0.01.

Transcript ID	Gene description	Fold change 24 hpi with WNV	Fold change 48 hpi with WNV
AALF021807	C-Type Lysozyme (Lys-E)	**433,71**	6,35
AALF003252	Unspecified product	**141,9**	19,98
AALF016234	C-Type Lectin	**82,89**	**17,75**
AALF010414	Beta-hexosaminidase b	**73,17**	1,87
AALF026511	Carbonic anhydrase	**70,41**	1,3
AALF013298	Unspecified product	**69,1**	**14,16**
AALF022426	Unspecified product	**58,22**	14,16
AALF009966	Unspecified product	**57,53**	4,39
AALF003774	Fibrinogen and fibronectin	**52,23**	**18,48**
AALF019859	Clip-Domain Serine Protease family B	**49,51**	**9,26**
AALF008821	Defensin anti-microbial peptide	**45,85**	**57,72**
AALF008776	Aromatic amino acid decarboxylase	**44,86**	2,51
AALF022425	Unspecified product	**30,38**	**2,29**
AALF009414	Putative secreted protein	**27,47**	4,74
AALF026731	Unspecified product	**22,22**	**98,88**
AALF001194	Unspecified product	**21,13**	5,21
AALF008229	Unspecified product	**19,82**	**7,04**
AALF001093	Calreticulin	**18,76**	-1,57
AALF007742	Unspecified product	**18,4**	2,27
AALF020341	Unspecified product	**18,35**	3,91
AALF023135	Unspecified product	**18,07**	3,07
AALF016505	Leucine-rich immune protein	**16,83**	2,96
AALF025212	Transferrin	**15,95**	**20,69**
AALF020197	Clip-Domain Serine Protease family B	**15,25**	-1,18
AALF020799	Peptidoglycan Recognition Protein	**14,48**	**10,23**
AALF000812	Unspecified product	**14,36**	1,98
AALF001195	Unspecified product	**12,68**	**9,79**
AALF007525	Unspecified product	**12,62**	4,11
AALF008452	Unspecified product	**12,07**	**10,83**
AALF003040	Unspecified product	**11,56**	2,87
AALF015567	Cystathionine beta-synthase	**11,47**	2,02
AALF014689	Fibrinogen and fibronectin	**10,71**	1,91
AALF002418	Imaginal disc growth factor	**10,45**	**10,37**
AALF023540	Unspecified product	**10,41**	-1,07
AALF001232	Pyruvate carboxylase	**10,24**	-3,08
AALF016365	Munc13-4	**10,04**	1,74
AALF011706	Unspecified product	**-10,8**	1,09
AALF028505	Unspecified product	**-13,71**	**-12,83**
AALF020168	Unspecified product	**-13,92**	-1,24

AALF025541	Unspecified product	**-15,28**	1,39
AALF008099	Endothelin-converting enzyme	**-15,38**	-3,91
AALF000742	Unspecified product	**-15,83**	1,29
AALF008879	Type IV inositol 5-phosphatase	**-16,64**	**-10,21**
AALF016756	Sugar transporter	**-16,64**	1
AALF022022	cdkl1/4	**-23,57**	-1,52
AALF004114	No-mechanoreceptor potential a	**-24,63**	1,32
AALF011390	Putative ecdysone-induced protein	**-26,46**	-2,2
AALF020798	Unspecified product	**-29,42**	-1,31
AALF000142	Unspecified product	**-31,2**	-2,65
AALF009909	Unspecified product	**-32,38**	-2,74
AALF001259	Unspecified product	**-36,11**	-1,03
AALF014478	Heat shock protein HSP70	**-37,19**	4,22
AALF012770	Aldehyde oxidase	**-37,85**	-2,04
AALF001105	Unspecified product	**-40,85**	1,86
AALF002857	Unspecified product	**-42,79**	-4,51
AALF002636	Unspecified product	**-43,23**	1,03
AALF014479	Unspecified product	**-45,7**	6,53
AALF025810	Unspecified product	**-51,7**	-1,85
AALF013937	Serine protease	**-51,98**	-2,31
AALF014375	Unspecified product	**-57,5**	-3,56
AALF014481	Unspecified product	**-61,02**	5,22
AALF027745	Microtubule-associated protein	**-61,92**	1,59
AALF009202	C-Type Lectin (CTL)	**-65,35**	1,56
AALF006472	Unspecified product	**-86,05**	-3,82
AALF015015	Unspecified product	**-230,7**	**22,72**
AALF015014	Clip-Domain Serine Protease family D	**-246,26**	-2,96
AALF016295	Unspecified product	**-249,48**	-5
AALF014395	Unspecified product	**-605,5**	-4,51
AALF028496	Unspecified product	**-78564,47**	1,33
AALF005731	Zinc finger protein	**-109162,03**	1,92
AALF009658	Unspecified product	-1,3	**31,17**
AALF019759	Unspecified product	1,81	**29,21**
AALF000656	Cecropin-A2	16,79	**27,72**
AALF010887	Unspecified product	-1,08	**23,96**
AALF011779	Unspecified product	3,84	**21,6**
AALF005659	Unspecified product	-1,38	**16,37**
AALF020693	Unspecified product	-2,54	**16,03**
AALF005658	Unspecified product	-3,45	**14,96**
AALF019136	Unspecified product	-1,68	**14,5**
AALF015016	Lethal(2)essential for life protein, l2efl	-9,24	**13,31**
AALF011176	Acylphosphatase	1,16	**13,16**
AALF000457	Unspecified product	1,46	**12,97**

AALF023579	Unspecified product	1,13	**12,72**
AALF026129	UPF0184 protein	1,93	**11,61**
AALF025560	c4b-binding protein beta chain	7,42	**11,43**
AALF014826	Unspecified product	1,78	**11,12**
AALF013319	Unspecified product	4,99	**11,12**
AALF018157	Unspecified product	5,32	**10,37**
AALF002626	40S ribosomal protein S21	-1,5	**10,19**
AALF006531	Unspecified product	6,54	**10,18**
AALF016228	Dolichol-phosphate mannosyltransferase subunit 3	2,49	**10,14**
AALF015599	Unspecified product	3,03	**-10,3**
AALF015221	Unspecified product	1,97	**-10,34**
AALF020651	Unspecified product	2,84	**-10,51**
AALF014559	Unspecified product	5,02	**-10,53**
AALF021818	Unspecified product	-1,64	**-10,76**
AALF016237	Unspecified product	2,97	**-11,03**
AALF024446	Unspecified product	3,83	**-11,82**
AALF015711	Unspecified product	**6,43**	**-12,13**
AALF000130	Unspecified product	3,56	**-12,54**
AALF019880	Unspecified product	1,25	**-12,85**
AALF009765	Unspecified product	-1,77	**-12,93**
AALF013917	Unspecified product	5,38	**-13,07**
AALF013610	Unspecified product	1,27	**-13,14**
AALF020871	Unspecified product	3,25	**-13,26**
AALF022735	Unspecified product	**5,48**	**-13,63**
AALF006220	Unspecified product	2,6	**-13,64**
AALF017380	Unspecified product	4,29	**-14**
AALF013129	Unspecified product	-1,93	**-14,88**
AALF006549	Unspecified product	3,88	**-15,21**
AALF022033	Unspecified product	1,04	**-15,3**
AALF018633	Unspecified product	-3,16	**-16,39**
AALF008765	Unspecified product	-1,18	**-16,52**
AALF025208	Unspecified product	4,68	**-17,47**
AALF013096	Unspecified product	1,63	**-19,32**
AALF020652	Unspecified product	3,74	**-19,54**
AALF000129	Unspecified product	4,63	**-20**
AALF021783	Unspecified product	7,45	**-22,85**
AALF013722	Unspecified product	5,27	**-24,51**
AALF013353	Unspecified product	6	**-29,13**
