# Supplementary figures and images for: Transcriptome Analysis of an Aedes albopictus Cell Line Single- and Dual-Infected with Lammi Virus and WNV
Source: Int J Mol Sci. 2022 Jan 14;23(2):875. doi: 10.3390/ijms23020875 (PMC8777793; doi:10.3390/ijms23020875)

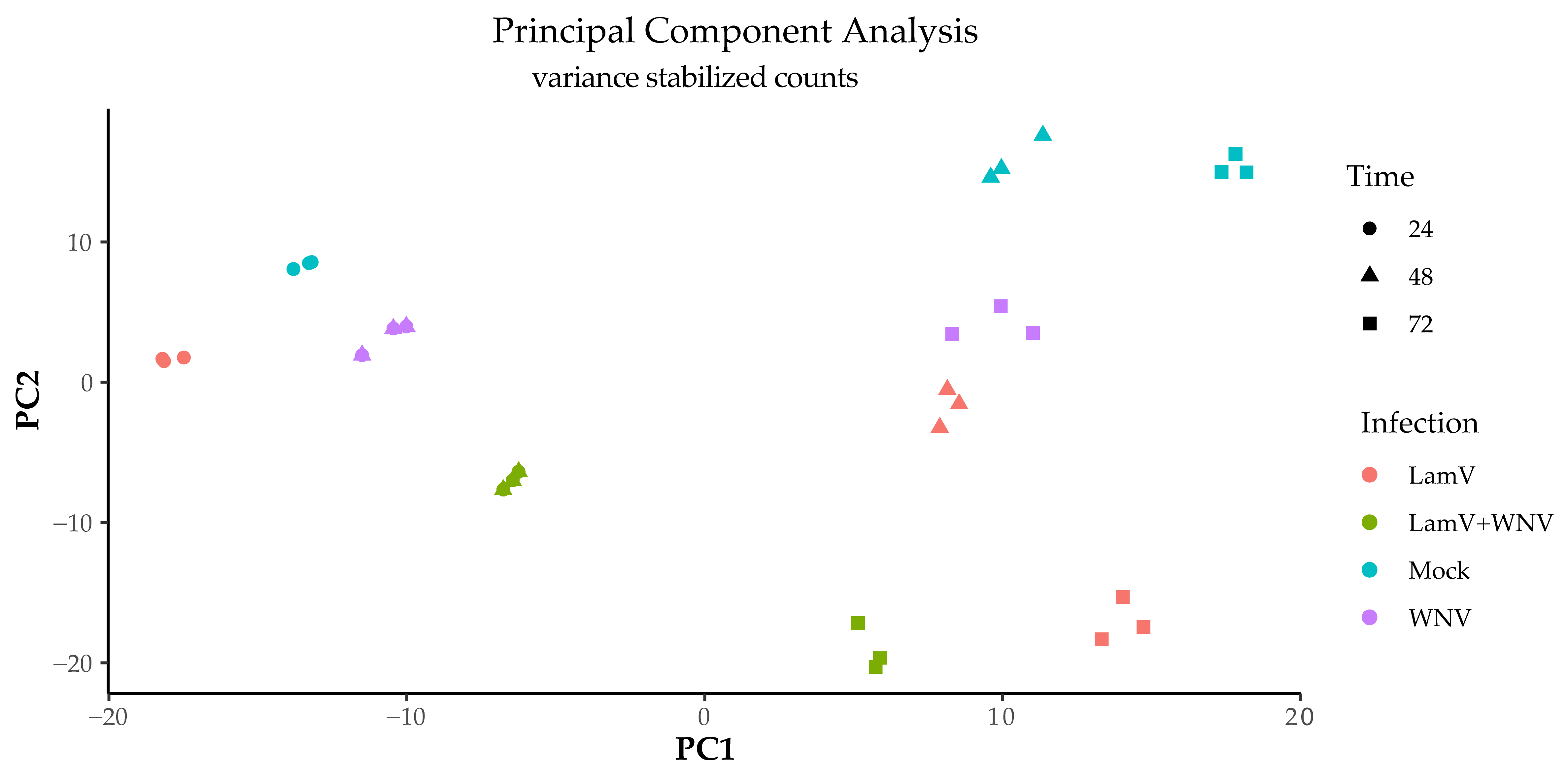

Supplement: Supplementary file 1 [file ijms-23-00875-s001.zip › Figure_S1.png]
